# Predictors of early death and clinical features in newly diagnosed patients with low-intermediate risk acute promyelocytic leukemia

**DOI:** 10.3389/fonc.2022.895777

**Published:** 2022-09-14

**Authors:** Jingjing Wen, Fang Xu, Qiaolin Zhou, Lin Shi, Yiping Liu, Jing Yue, Ya Zhang, Xiaogong Liang

**Affiliations:** Department of Hematology, Mianyang Central Hospital, School of Medicine, University of Electronic Science and Technology of China, Mianyang, China

**Keywords:** acute promyelocytic leukemia, low and intermediate risk, arsenic trioxide, early death, clinical characteristics, prognosis

## Abstract

**Background:**

Although most acute promyelocytic leukemia(APL) with low-intermediate risk could survive the induction treatment, early death still a big problem to have effects on overall survival in real world.This study aimed to analyze the clinical characteristics and possible predictors of early death in newly diagnosed patients with low-intermediate-risk acute promyelocytic leukemia.

**Methods:**

Sixty patients with newly diagnosed low/intermediate-risk APL admitted to Mianyang Central Hospital from January 2013 to December 2021 were retrospectively analyzed.

**Results:**

Sixty patients with a median age of 46 years (range, 17-75 years) were included. Fourteen patients (23.3%) were in low-risk group, and 46 patients (76.7%) were in intermediate-risk group. Fourteen patients (23.3%) died during induction treatment. Five patients died of hemorrhage, 5 of severe infection and 4 of differentiation syndrome. Multivariate analysis showed that HGB <65g/L at diagnosis (OR=38.474, 95%CI: 2.648~558.923, *P*=0.008) during induction treatment was an independent risk factors for early death in low- intermediate risk APL patients. In survival group, all patients achieved complete remission, the time to achieve remission was 25.87 ± 5.02 days, the average ATO dosage was 0.16 ± 0.03 mg/kg/day. In univariate analysis, there was no statistically significant difference in time span for remission when ATO dosage was in the 0.11~0.16mg/kg/day range. Compared with patients with low-risk APL, those with intermediate-risk APL had higher white blood cell counts (at diagnosis, day 3, day 5 and peak), higher level of lactate dehydrogenase, higher percentage of bone marrow promyelocytes, more platelet transfusions during treatment, and more early deaths (*P*<0.05). The overall survival of intermediate-risk APL patients seemed worse than those with low-risk APL (χ=5.033, *P* =0.025).

**Conclusions:**

In patients with low-intermediate risk APL, HGB <65g/L at diagnosis was an independent risk factors for early death. Remission could still be achieved at low-dose ATO without affecting the required time for low-intermediate risk APL patients. Differences in clinical characteristics were found between low-risk and intermediate-risk APL. The intermediate-risk group had higher early mortality risk than the low-risk group.

## Introduction

Acute promyelocytic leukaemia (APL) has become a highly curable hematologic neoplastic disease with a 10-year overall survival (OS) rate of 93.9% (1), due to all-trans retinoic acid (ATRA) and arsenic trioxide (ATO). However, a population-based epidemiological study showed that the early death rate within 30 days can still be as high as 29% (2). Therefore, the prevention and control of early death may be the key to improving OS in APL patients. Prognostic stratification based on the Sanz criteria (3) showed that low-intermediate risk APL accounted for the majority (77%). However, low-intermediate risk APL patients are believed to have a lower possibility of early death than those with high risk. Few studies have reported related data, especially in real world settings. This study aims to examine the risk factors for early death during induction therapy in APL patients with low-intermediate-risk, in order to facilitate the identification and prevention of early death.

## Patients and methods

### Patients

The clinical data of all newly diagnosed APL patients hospitalized in the Hematology Department of Mianyang Central Hospital from January 2013 to December 2021 were collected. The diagnosis was in accordance with the Chinese guidelines for the diagnosis and treatment of APL (2018) (4). All enrolled patients were aged ≥ 16 years. Patients with high-risk, secondary leukemia, combined with other hematological diseases or other active malignancies (requiring treatment), and pregnant or lactating women were excluded. A total of 60 newly diagnosed low-intermediate risk patients were included. Medical records of all patients were reviewed in detail to obtain information regarding clinical characteristics, laboratory parameters, treatment, efficacy, early death and survival. Patient laboratory data included complete blood count, coagulation, lactate dehydrogenase (LDH) and renal function at diagnosis and during induction treatment. Bone marrow-related examinations included the percentage of bone marrow promyelocytes, immunophenotype by flow cytometry, and PML-RARα genotyping. This study was approved by the Ethics Committee of Mianyang Central Hospital.

### Treatment regimens and evaluation

All patients with suspected APL received oral ATRA immediately. After the diagnosis of APL was established, ATO combined with ATRA was administered as induction therapy. Chemotherapy (hydroxyurea, anthracyclines, cytarabine and homoharringtonine) was administered when the white blood cell (WBC) count was higher than 4×10^9^/L during induction therapy and decided by the clinician. Supportive care (4) for hemorrhage: Aggressive platelet transfusion support to maintain platelets ≥30~50×10^9^/L; fibrinogen replacement with cryoprecipitate and fresh frozen plasma to maintain a level >1500 mg/L and PT and PTT close to normal values. The diagnosis of remission and relapse was based on the literature (5). The level of infection was according to the Adverse Event Evaluation Criteria (CTCAE) 5.0.

### Definition of end points

Prognostic stratification was performed according to the Sanz criteria (3), with a WBC count ≤10×10^9^/L and a platelet (PLT) count ≥40×10^9^/L defined as low risk, and a WBC count ≤10×10^9^/L and a PLT count <40×10^9^/L defined as intermediate risk. Differentiation syndrome (DS) was diagnosed according to the definition of Frankel (6) in patients with the following symptoms and signs: respiratory distress, unexplained fever, weight gain >5kg, pleural effusion, pericardial effusion, hypotension and acute renal failure. DS was classified into mild and severe according to the literature (7). If there were 4 or more symptoms, the patient was classified with severe DS. If there were 2 or 3 symptoms, this was classified as mild DS. Early death was defined as death within the first 30 days of presentation (8). OS was defined as the time from diagnosis to death (event) or last follow-up. Follow-up was performed by telephone and outpatient medical records. The follow-up deadline was January 20, 2022, with a median follow-up of 26.7 (0-109.7) months.

### Statistical analysis

All statistical analyses were performed with SPSS version 26.0. Measurement data conforming to normal distribution were represented by ± standard deviation. Non-normal measurement data were expressed by the median (range). Categorical variables were analyzed by the Chi-square or Fisher exact test, and continuous variables were analyzed by the t-test or nonparametric test if not normally distributed. Multivariate analysis was carried out by logistic regression model. OS was analyzed by the Kaplan-Meier method, and the difference between the groups was determined by the log-rank test. Two-tailed *P*<0.05 was regarded as significant.

## Results

### Clinical characteristics of the patients

Sixty newly diagnosed APL patients (26 males and 34 females) were included in this study. The median age was 46 (17-75) years, 10 patients (6.7%) were over 60 years old. Fourteen patients (23.3%) had low-risk APL, and 46 patients (76.7%) had intermediate-risk APL. At diagnosis, the median WBC count was 1.65 (0.19-9.8)×10^9^/L, median hemoglobin (HGB) level was 77.5 (33-114) g/L, median PLT count was 17 (4-95)×10^9^/L, and median fibrinogen (FIB) level was 1.43 (0.17-5.35) g/L. PML-RARα was positive in all patients. Genotyping of PML-RARα was performed in 40 cases: 24 cases (60%) of Bcr-1 type, 4 cases (10%) of Bcr-2 type, and 12 cases (30%) of Bcr-3 type. Complete karyotyping was performed in 32 cases, and the most common additional chromosomal abnormality was +8 (6/32, 18.75%). Patients suffering from infection accounted for 85% (51/60), including 65% (39/60) with grade 3 and 18.3% (11/60) with grade 4 infection. Hemorrhage occurred in 85% (51/60) of patients. The main bleeding sites were skin 30% (18/60), and oral mucous 23.3% (14/60). And 48.3% (29/60) of patients had only one bleeding site, 26.7% (16/60) had two bleeding sites, and 10.0% (6/60) had three bleeding sites. DS occurred in 60% (36/60) of patients, 38.3% (23/60) with mild and 21.7% (13/60) with severe DS. During induction, a median of 6.5 (0-22) units of red blood cells, a median of 2 (0-12) therapeutic doses of platelets and a median of 425(0~7580) ml of plasma were transfused. All patients developed grade III-IV myelosuppression during treatment and achieved hematological remission after induction therapy. Seventeen patients were tested for PML-RARα by PCR before consolidation treatment, and the qualitative results were negative.

### Predictors of early death for *de novo* APL patients with low-intermediate risk

There were no significant differences in age, infection, hemorrhage, DS rate, PML-RARα genotyping, and percentage of bone marrow blast cells or promyelocytes between the early death group and survival group (*P*>0.05). No differences were found in WBC count, C-reactive protein, LDH and FIB between the early death group and survival group at diagnosis (*P*>0.05).

Early death occurred in 14 (23.3%) patients with low-intermediate risk APL, the causes of early death were intracranial hemorrhage (5 patients), severe infection (5 patients) and DS (4 patients). Eight (57.1%) of these patients died within 4 days. In the early-death group, 1(0-3) therapeutic doses of platelets and 385 (0-2240) ml of plasma were transfused in the first 4 days. In the survival group, 1(0-3) therapeutic doses of platelets and 325(0-2680) ml of plasma were transfused in the first 4 days. No statistical differences of platelet (Z=0.038, P=0.970) and plasma (Z=0.915, P=0.360) transfusions were detected. Moreover, at death in 42.9% patients platelet count was more than 30×10^9^/L and in 64.3% patients FIB level was more than 1.5 g/L.

Compared with the survival group, the early death group had more male patients, more with intermediate-risk APL, earlier occurrence of DS, more severe DS, higher WBC count (day 3), lower HGB (at diagnosis), lower PLT count (at diagnosis, day 3, day 5 and chemotherapy), more patients with elevated creatinine at diagnosis and high cystatin C at chemotherapy, fewer patients treated with combination chemotherapy, and more transfusion usages of average daily plasma, all with statistically significant differences (*P*<0.05, **Table 1**). The cut-off value of WBC, HGB and PLT were determined by ROC curve.

**Table 1:** Univariate analysis of early death in patients with low-intermediate-risk APL.

Clinical characteristics	Early death group(n=14 cases)	Survival group(n=46 cases)	Statistical value (χor t or Z value)	*P-value*
Gender			9.234	0.002
male	78.6% (11/14)	32.6% (15/46)		
female	21.4% (3/14)	67.4% (31/46)		
Combination chemotherapy			10.825	0.001
yes	35.7% (5/14)	84.8% (39/46)		
no	64.3% (9/14)	15.2% (7/46)		
BMI (kg/m^2^)	23.75 ± 2.93	23.38 ± 3.55	0.223	0.825
Risk stratification			NA	0.026
low-risk	0% (0/14)	30.4% (14/46)		
intermediate-risk	100% (14/14)	69.6% (32/46)		
Severity of DS			4.720	0.030
mild	16.7% (1/6)	73.3% (22/30)		
severe	83.3% (5/6)	26.7% (8/30)		
The occurrence time of DS (days from APL diagnosis)	3.5 (3~12)	8 (3~19)	1.965	0.049
HGB at diagnosis (g/L)	65.07 ± 18.83	78.85 ± 17.68	2.515	0.015
≥65 g/L	13.3% (6/45)	86.7% (39/45)	7.950	0.005
<65 g/L	53.3% (8/15)	46.7% (7/15)		
PLT at diagnosis (×10^9^/L)	10.5 (4~32)	18 (4~95)	2.581	0.01
≥13×10^9^/L	11.9% (5/42)	88.1% (37/42)	8.203	0.004
<13×10^9^/L	50.0% (9/18)	50.0% (9/18)		
Creatinine (mmol/L)	77.4 (57.4~537.5)	59 (38.8~129.9)	2.746	0.006
WBC on Day 3 (×10^9^/L)	14.84 (1.00~22.83)	3.17 (0.43~29.61)	2.564	0.01
≥7×10^9^/L	33.3% (7/21)	66.7% (14/21)	NA	0.006
<7×10^9^/L	3.3% (1/30)	96.7% (29/30)		
PLT on Day 3(×10^9^/L)	14(2~31)	28(7~90)	2.449	0.014
PLT on Day 5 (×10^9^/L)	19 ± 4.38	45.6 ± 19.16	7.893	0.000
PLT at chemotherapy (×10^9^/L)	12 (5~23)	36 (9~134)	3.201	0.001
Cystatin C at chemotherapy (mg/L)	3.25 (1.71~4.78)	0.93 (0.7~2.29)	2.090	0.037
Transfusion of average daily PLT (therapeutic dose)^†^	0.12 (0-1)	0.07 (0-0.40)	0.702	0.483
Transfusion of average daily plasma (ml)^†^	141.40 (0-583.33)	16.65(0-379)	3.184	0.001

APL, acute promyelocytic leukemia; ATO, arsenic trioxide; WBC, white blood cells; HGB, hemoglobin; PLT, platelets; DS, differentiation syndrome; BMI, Body mass index; NA, not available. ^†^time span was defined as the day admitted to the hospital to death or remission.

Univariate analysis showed that the proportion of early death with WBC count≥7×10^9^/L on day 3 was significantly higher than that of the patients with WBC count <7×10^9^/L on day 3 (P<0.05). Moreover, a higher proportion of early death in patients with HGB<65g/L or PLT<13×10^9^/L than that in patients with HGB≥65g/L or PLT≥13×10^9^/L, the difference were statistically significant (**Table 1**).

Multivariate analysis was used to examine the effects of gender, WBC≥7×10^9^/L at diagnosis, HGB <65 g/L at diagnosis, PLT<13×10^9^/L at diagnosis, DS and combination chemotherapy on early mortality. It was shown that HGB <65g/L at diagnosis (OR=38.474, 95%CI: 2.648~558.923, *P*=0.008) was an independent risk factor for early death during induction treatment.

In early death group, only 5 patients were available for analysis of ATO dosage, the average ATO dosage was 0.14 ± 0.04 mg/kg/day. In survival group, all patients achieved complete remission, the time to achieve remission was 25.87 ± 5.02 days, the average ATO dosage was 0.16 ± 0.03 mg/kg/day which was not statistically different from the ATO dosage of the death group (*t*=1.072, *P*=0.289). Univariate analysis showed that patients with ATO dosage ≥0.16 mg/kg/day needed a slightly longer time to achieve remission during induction therapy than patients with ATO dosage <0.16 mg/kg/day(26.59 ± 5.18 vs.24.71 ± 4.45 days), but the difference wasn’t statistically significant(*t*=1.271,*P*=0.211). Similar results could be seen when ATO dosage(mg/kg/day) thresholds were 0.11, 0.12, 0.13, 0.14, 0.15, and the P value was 0.844, 0.174, 0.220, 0.166, 0.492, respectively.

### Comparison of clinical features between low and intermediate risk APL patients

Early death rate was higher in intermediate-risk patients than in low-risk patients (30.4% vs. 0.0, *P*=0.026). Patients with intermediate-risk required more platelet transfusions during treatment than those in the low-risk group (*P*=0.001). The WBC count at diagnosis, day 3, day 5 and peak were all higher in the intermediate-risk group than in the low-risk group (*P*=0.038, 0.001, 0.001, 0.017, respectively). The percentage of monocytes at diagnosis (*P*=0.000) and day 3 (*P*=0.002) were both higher in the intermediate-risk group than in the low-risk group. Compared with the low-risk group, patients in the intermediate-risk group had higher LDH levels (*P*=0.008) and a higher percentage of bone marrow promyelocytes (*P*=0.016). The level of FIB in the intermediate-risk group was lower than that in the low-risk group, but the difference was not statistically significant (*P*=0.05) (**Table 2**).

**Table 2:** Comparison of clinical characteristics between low-risk and intermediate-risk APL patients.

Clinical characteristics	APL (n=60 cases)		Statistical value(Z)	*P-value*
	Low-risk (n=14)	Intermediate-risk (n=46)		
WBC at diagnosis (×10^9^/L)	0.96 (0.43~9.29)	2.20 (0.19~9.8)	2.071	0.038
Percentage of monocytes at diagnosis (%)	8.65 (2~42.3)	32.5 (8.2~83.8)	3.540	0.000
LDH (U/L)	214.5 (117~315)	273 (131~2802)	2.670	0.008
FIB (g/L)	2.07 (0.17~5.35)	1.31 (0.27~3.63)	1.956	0.050
WBC on day 3 (×10^9^/L)	1.08 (0.43~11.84)	7.21 (0.56~29.61)	3.437	0.001
Percentage of monocytes on day 3 (%)	5.8 (1.1~39.6)	23.2 (5.0~91.3)	3.031	0.002
WBC on day 5 (×10^9^/L)	1.30 (0.24~26.69)	13.19 (0.49~73.73)	3.208	0.001
Peak of WBC (×10^9^/L)	18.18 (1.56~40.1)	22.83 (3.43~110.58)	2.381	0.017
Percentage of bone marrow promyelocytes (%)	69 (46~92.5)	83.5 (26~96)	2.406	0.016
Transfusion of PLT (therapeutic dose)	0 (0~2)	2 (0~12)	3.398	0.001

APL, acute promyelocytic leukemia; LDH, lactate dehydrogenase; FIB, fibrinogen; WBC, white blood cells; HGB, hemoglobin; PLT, platelets.

No significant differences were found in terms of gender, age, infection, hemorrhage, FIB, combination chemotherapy, DS occurrence, severity of DS, PML-RARα genotype distribution, RBC transfusion, HGB level at diagnosis, renal function and the time to achieve remission (*P*> 0.05) between the two groups.

### Overall survival

**Figure 1:**
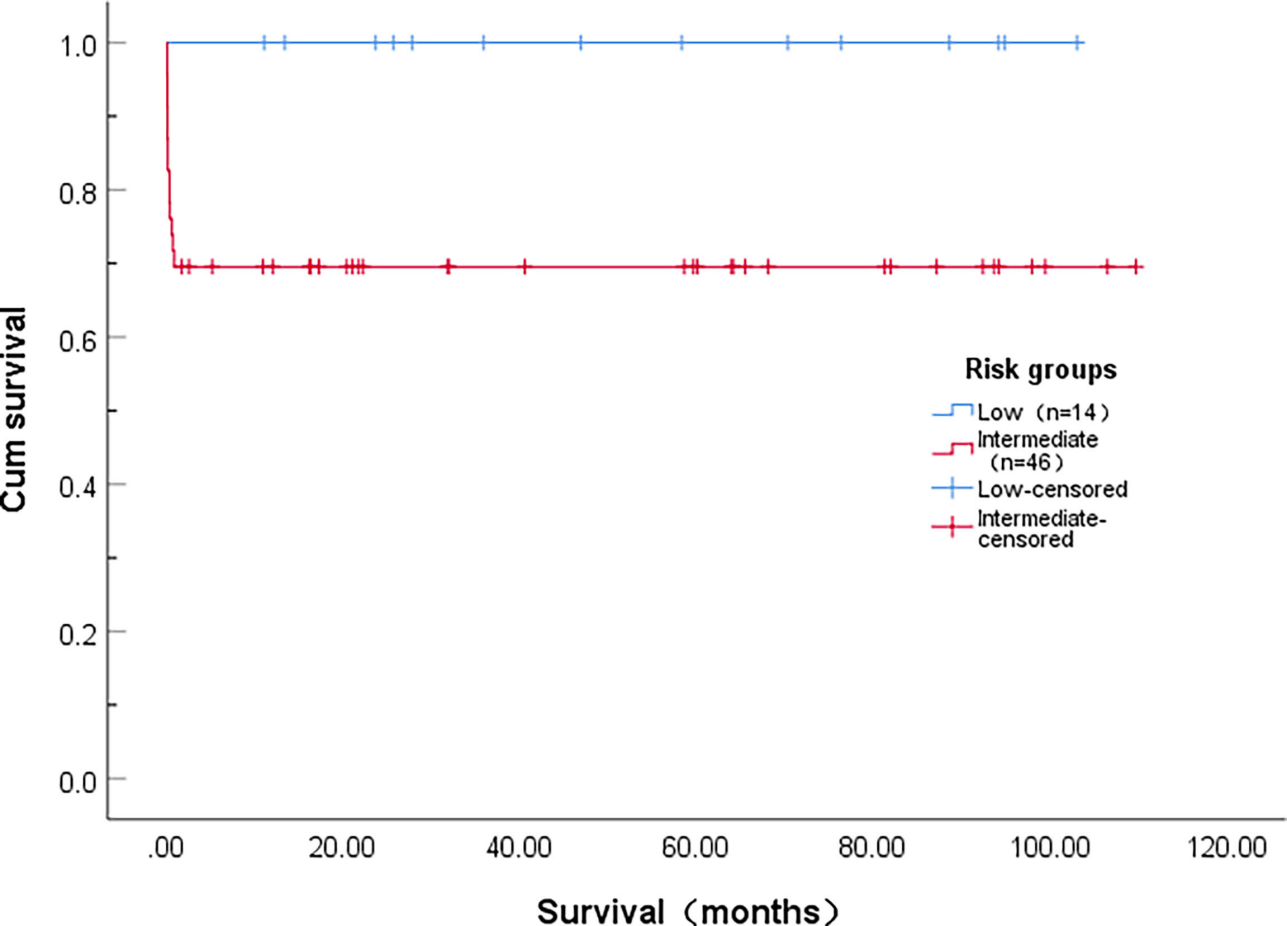
Overall survival of patients with acute promyelocytic leukemia stratified according to level of risk.

All patients who did not die early achieved long-term survival. Only one patient relapsed who did not follow the doctor’s instructions regarding maintenance therapy. The OS of intermediate-risk APL patients was significantly worse than that of low-risk APL patients (χ=5.033, *P*=0.025). The median OS was not achieved, 5-year OS rates in low-risk and intermediate-risk APL patients were 100% and 69.6%, respectively (**Figure 1**).

## Discussion

Compared to non-APL, APL patients are highly curable (9). But early death is still an important factor affecting the overall survival (10).The early death rate in our study was 23.3%, which was similar to that (22%-25%) reported in the literature (11), but higher than 16% reported by Harinder Gill et al. (12).In the present study, all admitted APL patients during the study period were included, which to some extent represented the real-world situation in our center. The results showed that the main causes of early death were intracranial hemorrhage, DS and infection, which were consistent with the previous report (12).

Our study showed that HGB <65g/L at diagnosis was an independent risk factor for early death. This suggests that patients with HGB <65 g/L at diagnosis have a high risk of early death, require more transfusions and closer observation. Fang Xu et al. (13) reported that APL patients with low-intermediate risk, and those in the early death group had lower HGB levels than survivors (*P*=0.015). However, they did not identify the effect of HGB level on early death using multivariate analysis. Silvia Park et al. (14) found that APL patients with HGB <8.0 g/dl had a lower complete remission rate than those with HGB ≥8.0 g/dl. These researchers designed a risk stratification model named the CBC-score based on HGB level, WBC count and PLT count. In terms of complete remission rate, early mortality and OS, the CBC-score seemed to have better power in predicting prognosis. This suggested that inclusion of HGB level in a risk stratification model may optimize the prediction of prognosis in APL patients. However, some results showed that there was no significant difference in HGB level between the early death group and survival group (12, 15). This was inconsistent with the results in our study, and may be related to the different data type and cut-off values of the dependent variables defined by the researchers. In addition, high-risk APL patients were not included in our study.

ATO induces apoptosis, autophagy of leukemia cells, and partial cellular differentiation as well as inhibition of cell growth and angiogenesis (16). The addition of ATO to APL induction therapy reduces relapse, prolongs survival and improves prognosis (17). Guidelines recommend that ATO should be administered at the dose of 0.15 to 0.16 mg/kg/d during induction therapy (4) ^(^ 18 ^),^.Shen et al. (19) reported on a low-dose (0.08 mg/kg d^−1^, for 28 days) As_2_O_3_ treatment for relapsed APL and there was no difference compared with those values in patients treated with a conventional dose. The authors concluded that low-dose As_2_O_3_ may have the same effect as the conventional dosage and the mechanism of low-dose arsenic seemed to primarily induce differentiation of APL cells. Yuan W et al. (20) evaluated the effects of low-dose ATO on differentiation *in vitro* using the embryonic stem cells of a mouse, and reported that low-dose ATO exposure would induce differentiation, other than apoptosis. However, newly diagnosed APL treated with ATO less than traditional dose was rarely reported. In our report, the time to remission in the low-dose ATO group(0.11~0.15mg/kg/d) was the same as that in the conventional-dose(0.16mg/kg/d) group. The time to achieve remission was 25.87 ± 5.02 days in our study which was consistent with 25.5days(range18~35) reported by Breccia M et al. (21). Moreover, all patients achieved complete remission in survival group. This suggested that remission could still be achieved at low-dose ATO without affecting the required time for low-intermediate risk APL patient.

As reported by Ciftciler R et al. (15), hemorrhage, infection, WBC count, PLT count, FIB, LDH, percentage of bone marrow blast cells, and risk stratification were independent risk factors for early death. A study (12) which included 358 newly diagnosed APL patients (aged 1-97 years) from Hong Kong showed that males, WBC count ≥10×10^9^/L and FIB <1.5 g/L were independent risk factors for early death. Yaxue Wu et al. (22) reported that age, WBC count, LDH and peripheral blood promyelocyte ratio in the early death of newly diagnosed APL patients were significantly higher than in patients who did not die early (*P*<0.05). However, multivariate analysis showed that only patients ≥50 years old and WBC count ≥10×10^9^/L were independent risk factors for early death in APL patients. Our report revealed that early death was more common in males, intermediate-risk patients, those with a higher WBC count and lower PLT count than in surviving patients. These factors were not identified as independent risk predictors for early death. Different from the above studies, in the present study no statistically significant differences in age, infection, hemorrhage, FIB, LDH, percentage of bone marrow blast cells and promyelocytes were found. All the above studies (12, 15, 22) mentioned that a WBC count ≥10×10^9^/L at diagnosis was an independent risk factor. Our study only focused on APL patients with low-to-intermediate risk. Patients with different risk stratification and different age span may account for the inconsistency in other independent risk predictors.

Our previous study (23) found that intermediate-risk APL patients had a faster WBC doubling time than low-risk patients. In the present study, compared with the low-risk group, the WBC count (at diagnosis, day 3, day 5, and peak) and the percentage of monocytes (at diagnosis and day 3) were higher in the intermediate-risk group. It indicates that the WBC growth rate in intermediate-risk APL is faster than that in low-risk APL. Moreover, LDH and the percentage of bone marrow promyelocytes were higher in the intermediate-risk group. This suggests that the tumor burden in intermediate-risk APL patients is significantly higher than that in low-risk APL patients. In addition, platelet transfusion was more common in the intermediate-risk group. Yaxue Wu et al. (24) reported that the rate of early death in APL patients with low, intermediate and high risk was 0, 4.5% and 22.7%, respectively. Early death rate of intermediate-risk APL patients was 30.4% in our study, higher than that reported by Yaxue Wu et al. (24). This may be related to more elderly patients included in our study, economic factors in patient’s families, and insufficient blood products support in the real world. The recommended supportive measures to treat the coagulopathy have not changed during the last decade and consist of generous transfusions of fresh frozen plasma and platelets to maintain the fibrinogen concentration and platelet count above 100–150 mg/dL and 30–50×10^9^/L, respectively (25). The usage of platelet and plasma transfusions during induction therapy was rarely reported. In our study, the median survival time of the early death group was 4 days, so we analyzed the transfusion amounts of platelets and plasma within 4 days of admission in the early death and survival groups to evaluate the transfusion support. No statistical differences of platelet and plasma transfusions were detected. The impact of other clinical markers on prognosis were investigated in APL. Shufen Li et al (26) reported that high BMI affected overall survival of APL patients but didn’t affect overall survival of AML patients, they suggested that high BMI may be predictor of adverse clinical outcomes in APL. In our study, BMI had no effect on early death.

In 2000, Sanz MA et al. (3) found that the relapse-free survival rate of newly diagnosed APL patients was significantly different among low, intermediate and high-risk groups. The Sanz prognostic scoring model is widely used. The Chinese guideline for the diagnosis and treatment of APL (2018) (4) stratified Sanz criteria into the guidelines, but treatment was divided into low-intermediate risk and high-risk groups. The NCCN Guidelines (2022version) (18) divided APL into low-risk and high-risk groups for stratified treatment according to WBC count. Our results revealed that the OS of intermediate-risk APL patients was still different from that in low-risk APL patients, which was similar to that reported by Sanz MA et al. (3) The early death rate in intermediate-risk APL patients was obviously higher than that in low-risk APL patients. This suggests that induction therapy in intermediate-risk APL patients should be individualized and different to that in low-risk APL patients, and more aggressive support is needed to improve long-term survival.

In conclusion, these findings suggest that HGB <65 g/L at diagnosis during induction treatment was an independent risk predictor for early death in newly diagnosed APL patients with low-intermediate risk. Therefore, adequate transfusion support should be emphasized to reduce the risk of early death in low-intermediate risk APL patients. In addition, remission could still be achieved at low-dose ATO without affecting the required time for low-intermediate risk APL patients. Despite a good OS in APL patients, there are still differences in clinical characteristics and prognosis between patients with low risk and intermediate risk. This suggests that induction treatment should be individualized in patients with different risk level. Due to our retrospective, single-center study design, and limitations due to missing data and small sample size, these conclusions require further validation by expanding the sample size.

## Data availability statement

The raw data supporting the conclusions of this article will be made available by the authors, without undue reservation.

## Ethics statement

The studies involving human participants were reviewed and approved by The Ethics Committee of Mianyang Central Hospital (Mianyang, China; approval no. S-2019-099). The patients/participants provided their written informed consent to participate in this study.

## Author contributions

JW and FX contributed to conception and design of the study. JW organized the database. JW and QZ performed the statistical analysis. LS, JY, YZ, YL, and XL interpreted the data and searched literature. JW wrote the first draft of the manuscript. FX and JW edited the manuscript. All listed authors approved the final manuscript.

## Conflict of interest

The authors declare that the research was conducted in the absence of any commercial or financial relationships that could be construed as a potential conflict of interest.

## Publisher’s note

All claims expressed in this article are solely those of the authors and do not necessarily represent those of their affiliated organizations, or those of the publisher, the editors and the reviewers. Any product that may be evaluated in this article, or claim that may be made by its manufacturer, is not guaranteed or endorsed by the publisher.

## Abbreviations

ATRA, all-trans retinoic acid; ATO, arsenic trioxide; APL, acute promyelocytic leukemia; DS, differentiation syndrome; WBC, white blood cell; HGB, hemoglobin; PLT, platelet; FIB, fibrinogen; LDH, lactate dehydrogenase; OS, Overall survival; RBC, red blood cell; BMI, body mass index.
